# Hemodynamics in diabetic human aorta using computational fluid dynamics

**DOI:** 10.1371/journal.pone.0202671

**Published:** 2018-08-23

**Authors:** Eunji Shin, Jung Joo Kim, Seonjoong Lee, Kyung Soo Ko, Byoung Doo Rhee, Jin Han, Nari Kim

**Affiliations:** 1 National Leading Research Laboratory for Cardiovascular Engineering, Department of Physiology, College of Medicine, Cardiovascular and Metabolic Disease Center, Inje University, Busan, South Korea; 2 Department of Internal Medicine, College of Medicine, Inje University, Seoul, South Korea; The Ohio State University, UNITED STATES

## Abstract

Three-dimensional (3D) computational aortic models have been established to reproduce aortic diseases such as aortic aneurysm and dissection; however, no such models have been developed to study diabetes mellitus (DM). To characterize biomechanical properties of the human aorta with DM, reconstructed aortic CT images were converted into DICOM format, and imported into the 3D segmentation using Mimics software. This resulted in a 3D reconstruction of the complete aorta, including three branches. We applied a pulsatile blood pressure waveform for the ascending aorta to provide a biomimetic environment using COMSOL Multiphysics software. Hemodynamics were compared between the control and DM models. We observed that mean blood flow velocity, aortic pressure, and von Mises stress values were lower in the DM model than in the control model. Furthermore, the range of aortic movement was lower in the DM model than in the control model, suggesting that the DM aortic wall is more susceptible to rupture. When comparing biomechanical properties in discrete regions of the aorta, all values were higher in the ascending aorta for both control and DM models, corresponding to the location of most aortic lesions. We have developed a compute based that integrates advanced image processing strategies and computational techniques based on finite element method to perform hemodynamics analysis based on CT images. Our study of image-based CFD analysis hopes to provide a better understanding of the relationship between aortic hemodynamic and developing pathophysiology of aortic diseases.

## Introduction

Diabetes mellitus (DM) is a highly prevalent disease leading to increased morbidity and mortality [1, 2]. DM is a major risk factor for cardiovascular disease and is associated with several micro- and macro-vascular complications including diabetic nephropathy retinopathy, neuropathy, coronary artery disease, peripheral arterial disease, and stroke [3–7]. Because the prevalence of DM continues to increase in developed and developing countries [8–10], it is important to understand the relationship between DM and vascular alterations. Several studies suggest that endothelial dysfunction, oxidation, inflammation, and vascular remodeling play key roles in the development of angiopathies in both animal models of DM and DM patients [11–13]. However, whether DM-associated hemodynamic changes affect human aortic properties remains unknown.

Although many hemodynamic simulation studies have already been conducted, including those modeling aneurysm, dissection, and atherosclerosis of the aorta [14–16], no studies have yet simulated DM-related changes in blood and aortic wall properties. To compare control and DM aortic models during the cardiac cycle, we developed a computational fluid dynamic finite element model that simplifies complex anatomy in a biomimetic environment. Then, three basic parameters to analysis the hemodynamic phenomenon were obtained using computational fluid dynamic method: velocity, pressure and von Mises stress. Velocity allows yielding wall shear stress that plays a key role in the hemodynamic analysis of atherosclerotic model [17]. In addition, the pressure distribution and von Mises stress are used in the wall mechanics of aorta aneurysm and associated rupture potential [18, 19]. Our DM aortic model mimics high viscosity and stiffness conditions, allowing these properties to be linked to changes in blood flow and alterations of the aortic wall. Thus, the results of this modeling study could be clinically applied to improve the management and prognosis of DM patients.

## Materials and methods

### Human subject

A human aorta (50-year-old male) was selected anonymously in this study to gather the CT scan (Siemens Medical Solutions, Erlangen, Germany) data for generating a 3D aorta model. Patient information and CT images were obtained from the Department of Cardiology, Seoul National University Hospital on April 21, 2016. The participant provided written informed consent. The study protocol was approved by the Seoul National University Ethics Committee (IRB No. H-1601-132-739). None of the tissue donors were from a vulnerable population and all donors or next of kin provided written informed consent that was freely given.

### Three-dimensional (3D) aortic geometry and meshing

The CT scan images were obtained with a 128-multislice scanner with the tube voltage of 120 kV. The images of the patient were saved in a DICOM (Digital Imaging and Communications in Medicine) file, and a 3D real aorta model was reconstructed using this CT data with the MIMICS 19 software. Segmentation of the data was performed to define a range of thresholding values to obtain a segmentation mask in the MIMICS software. In this study, all aortic models have different thresholds. After setting the optimized thresholds, a region growing function for each threshold was adjusted to create the appropriate aorta mask, and the unwanted mask was manually modified. The aorta model was saved in a stereolithography (STL) file format. The 3D surface model of the aorta was imported into the 3-matic meshing software for smoothing. Geometric modeling of the aorta was performed using the SpaceClaim under Ansys Workbench 18 software.

A mesh containing 119,666 tetrahedral elements, 57,008 prism elements, and 19,450 triangular elements were established and refined to maximize the accuracy and reliability of the model solutions. We set the boundary layer value to 3, the boundary layer stretching factor to 1.2, and the thickness adjustment factor to 5. The image-based geometry of the aorta consisted of the ascending aorta, aortic arch, descending aorta, brachiocephalic artery, left common carotid artery, and left subclavian artery.

### Material properties

We assumed that blood was an incompressible Newtonian fluid with a constant viscosity. The blood boundary was set to a no-slip condition, and zero viscous stress was applied at the inlet [20, 21]. We assumed that the aortic wall was an isotropic rigid solid that was non-permeable with no slip. The Young’s modulus, Poisson’s ratio, and density were used to define the properties of the aortic wall [22–24]. The different material properties applied in this study between control and DM model is viscosity and Young’s modulus. Dynamic viscosity of blood were taken to be 0.00350 Pa·s and 0.00596 Pa·s in control and DM model, respectively. Young’s modulus of aortic wall applied in control and DM model were 7.18MPa and 9.93MPa, respectively. We constrained the inlet and outlet of the aortic wall to not move with fixed constraints.

### Boundary conditions

**Fig 1:**
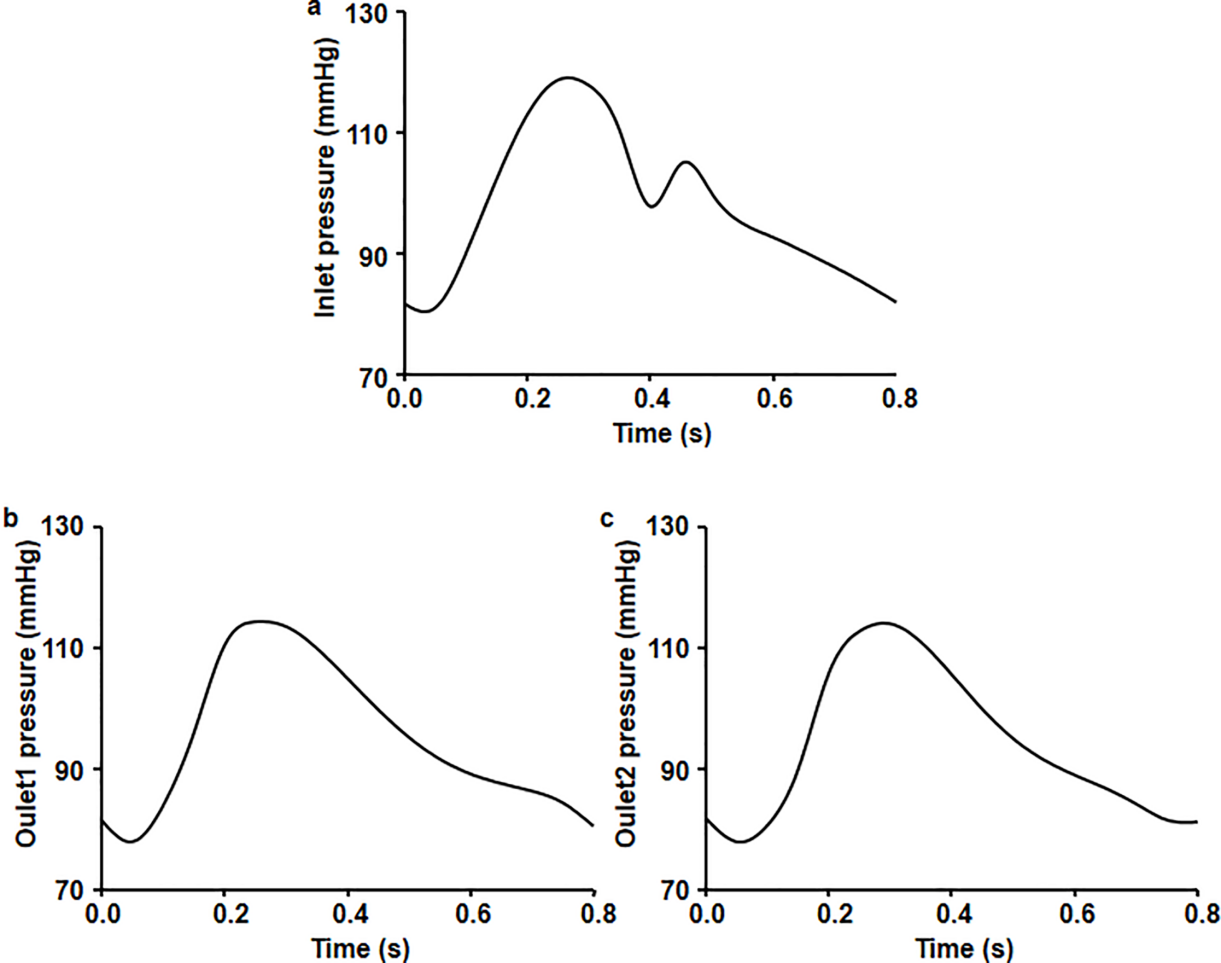
Boundary condition pressure waveforms imposed at one inlet (ascending aorta root) and two outlets (branches and descending aorta region). Blood pressure at (a) the inlet of the ascending aorta, (b) the exit of the branches, and (c) the descending aorta.

As blood pressure at the ascending aorta is usually available in the form of a pulsatile normal blood pressure waveform *in vivo*, we applied a pulsatile normal blood pressure waveform from literature for the ascending aorta to provide a biomimetic environment. We also applied boundary conditions at the inlet and outlets (Fig 1) [25].

### Computational fluid dynamics

The set of governing equations along with the boundary conditions were solved by COMSOL Multiphysics v.5.2a with finite element methods.

$$\rho \frac{\partial u}{\partial t}+\rho (u∙\nabla )u=\nabla ∙[-pI+\mu (\nabla u+{\left(\nabla u\right)}^{T})]+F,\;\rho \nabla ∙u=0$$

This equation was solved by iterative, advanced and fully coupled solvers. Time-dependent simulations were performed on a computer with a 2.60 GHz Xeon(R) CPU E5-2697 v3 with 128 GB of RAM.

### Analysis

3D aortic model simulations were run for 81 steps because we noted a convergence in blood flow cycle at 0.8 s and 0.01 s intervals. Dependent measures from the simulations included blood flow, aortic pressure, and pressure-induced stress. Differences in the performance of the aorta across the cardiac cycle between the ascending and descending aorta and between the control and DM models were analyzed.

## Results

**Fig 2:**
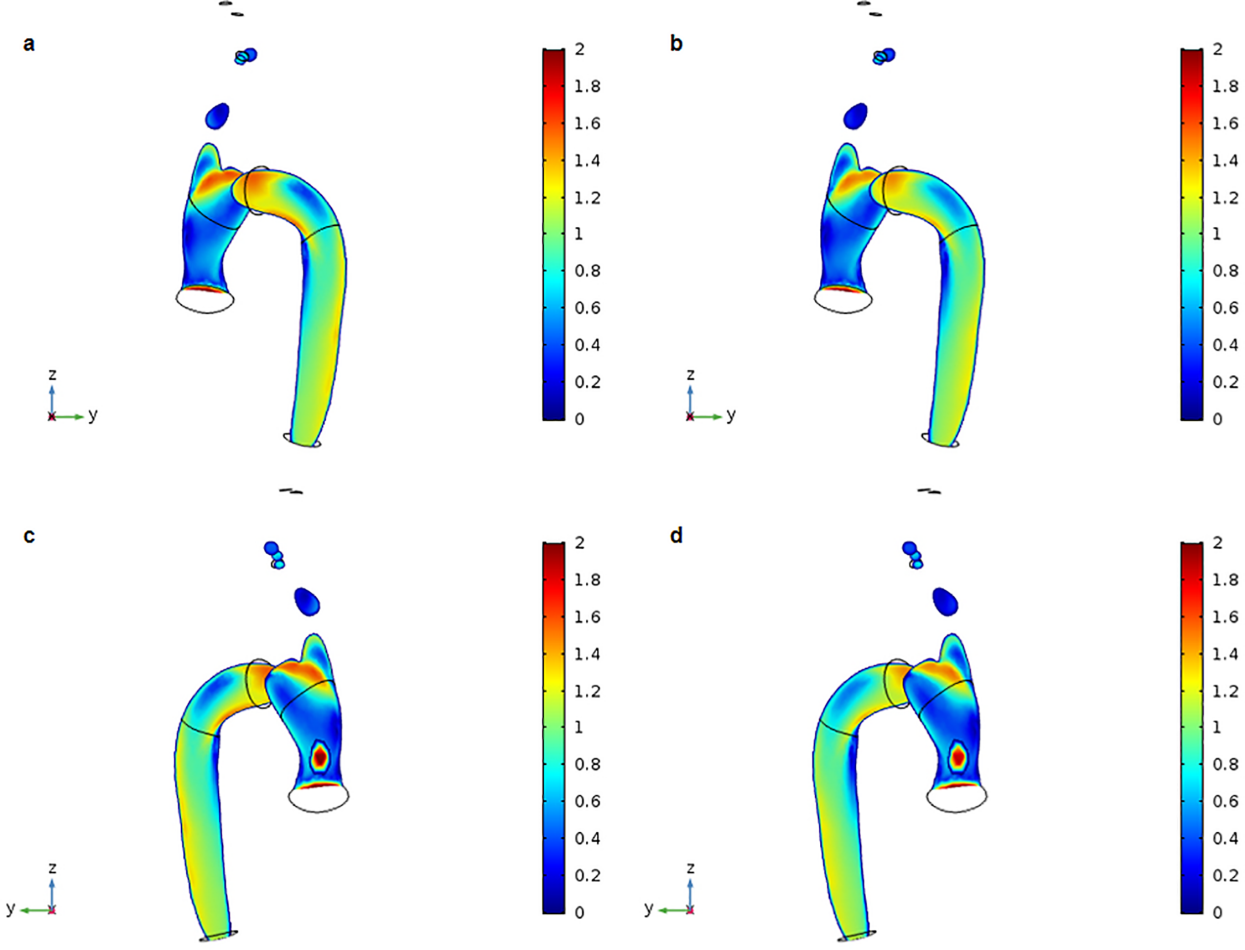
3D distribution of blood flow velocity at the systolic peak (t = 0.27 s). (a) Control aortic model divided into y-z axial slices. (b) DM aortic model divided into y-z axial slices. (c) Control aortic model other side divided by y-z axial slices. (d) DM aortic model other side divided by y-z axial slices.

**Fig 3:**
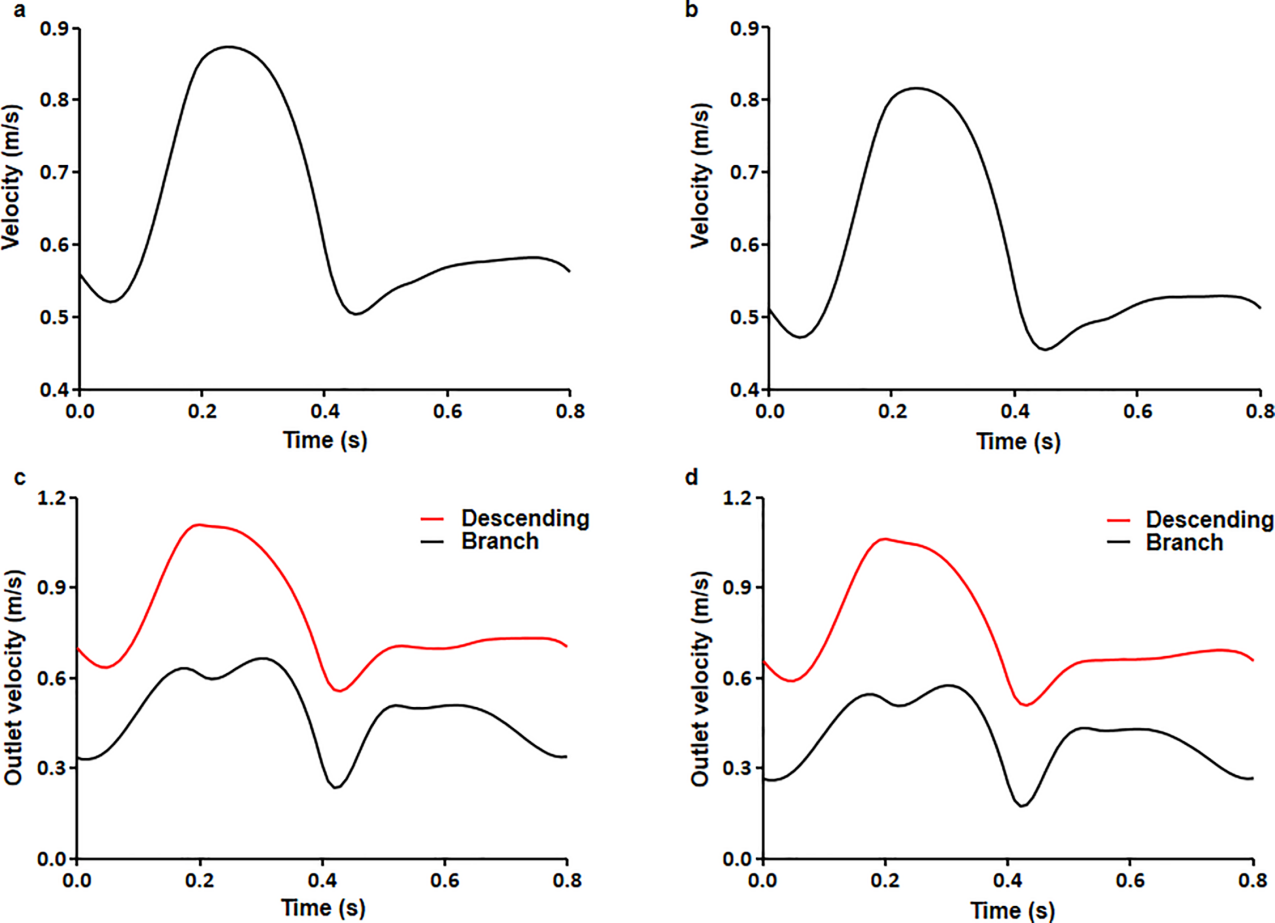
Overall mean blood flow velocity. (a) Control and (b) DM aortic models during the cardiac cycle. Mean blood flow velocities at the outlet of the branches and the descending aorta for the (c) control and (d) DM aortic model.

The distributions of blood flow velocity at peak systole under normal pressure for the control and DM aortic models are shown (Fig 2). In the ascending aorta, the maximum flow velocity occurred toward the outer wall of the proximal aortic arch, and flow velocity gradually decreased toward the inner wall of the aortic arch. Flow velocity also increased moving downstream into the descending aorta due to tapering. Overall, there was a difference in mean flow velocity between the outer and inner walls of the aorta, with faster flow velocity near the outer wall than near the inner wall. Furthermore, mean flow velocity values were slightly higher in the control model (0.637 m/s) than in the DM model (0.585 m/s). Time-dependent flow velocity values for control and DM models under normal physiological conditions were calculated. Flow velocity ranged from 0.503–0.873 m/s for the control model and 0.454–0.816 m/s for the DM model (Fig 3A and 3B). Flow velocity was higher at the outlet of the descending aorta than at the branches. Furthermore, flow velocity at the outlet of the descending aorta was lower in the DM model than in the control model (Fig 3C and 3D).

**Fig 4:**
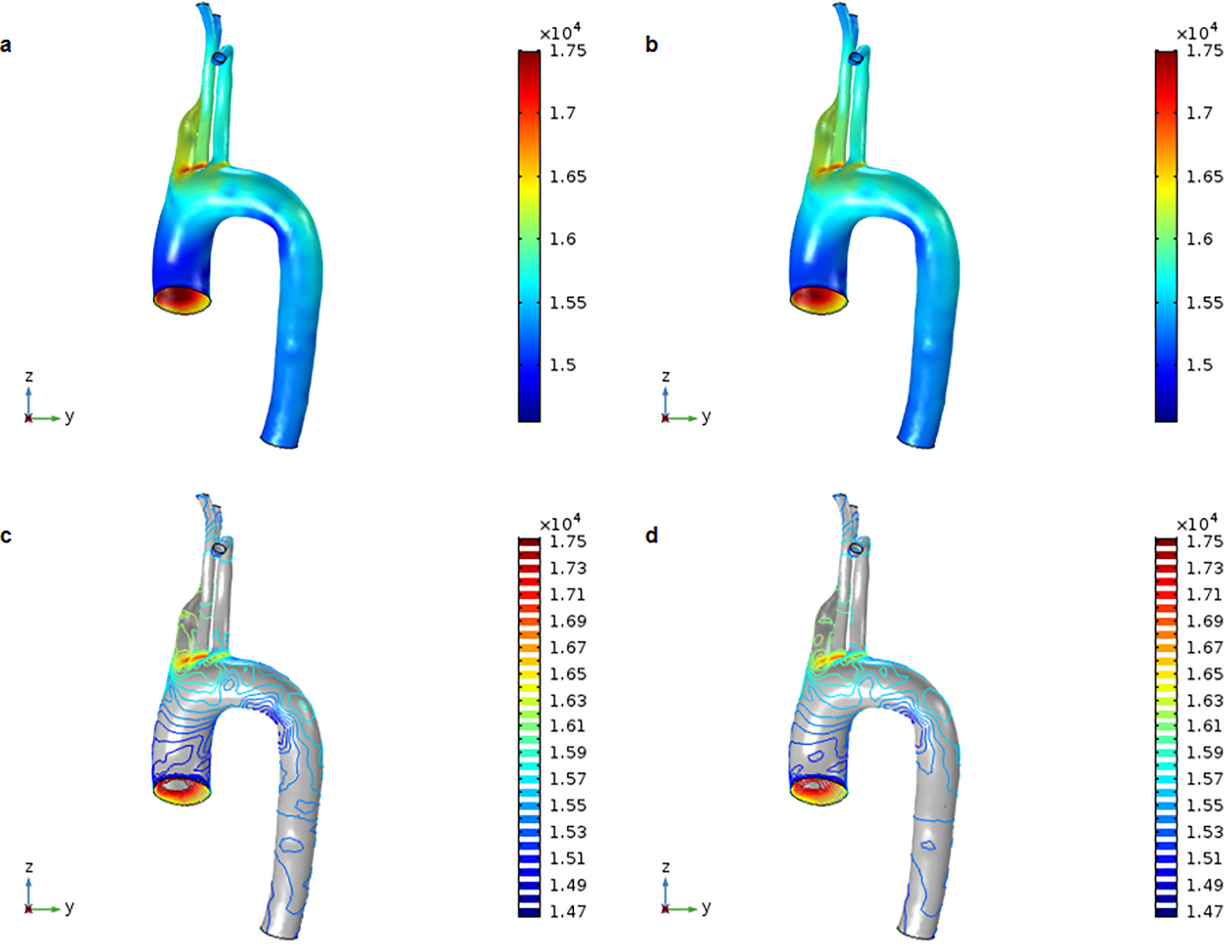
3D distribution of blood pressure at the systolic peak (t = 0.27 s). (a) Surface values for the control aortic model. (b) Surface values for the DM aortic model. (c) Contour values for the control aortic model. (d) Contour values for the DM aortic model.

**Fig 5:**
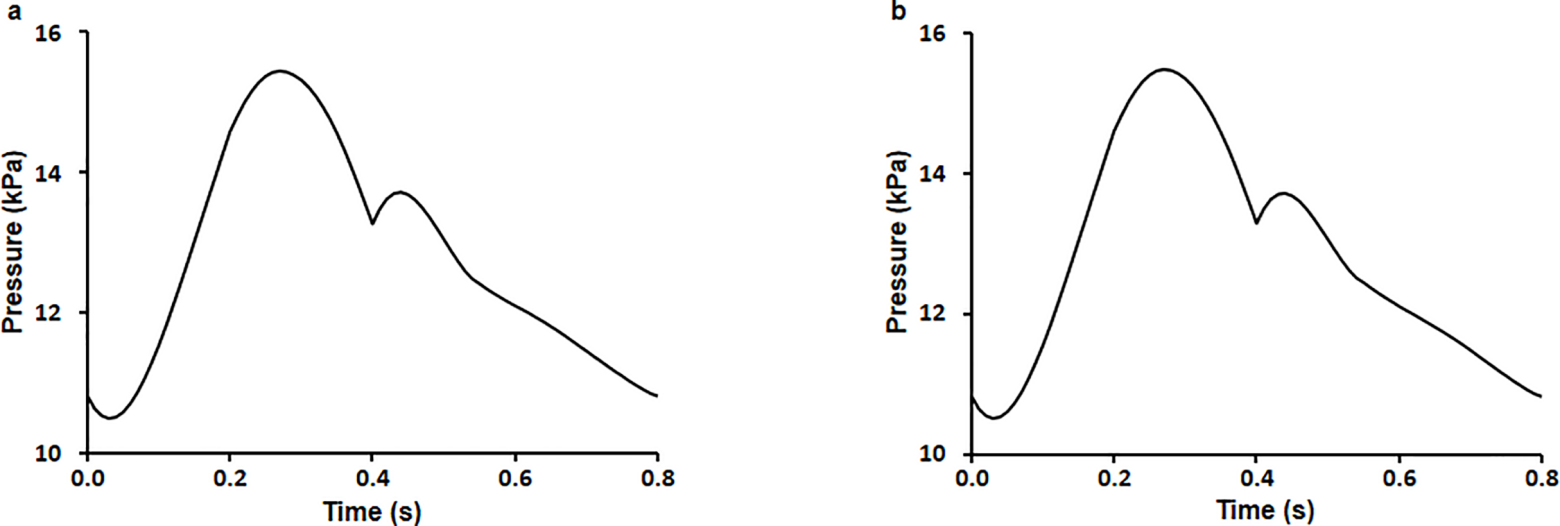
Overall mean blood pressure. (a) Control and (b) DM aortic models during the cardiac cycle.

Blood pressure in the control aortic model was higher than that in the DM aortic model. The maximum and minimum peak systolic pressures were 17.746 and 14.558 kPa, respectively, for the control model, and 17.500 and 14.673, respectively, for the DM model. Blood pressure tended to be higher in the ascending aorta than in the descending aorta as a result of the slower velocity of pumped blood as it approached the aortic arch due to its curvature (Fig 4). There was little difference between the models in the mean pressure of the whole aorta, which ranged from 10.501–15.452 kPa for the control model and 10.518–15.497 kPa for the DM model over time (Fig 5). Therefore, although flow velocity was shown to vary depending on the location of the aorta, we found that blood pressure was distributed more evenly.

**Fig 6:**
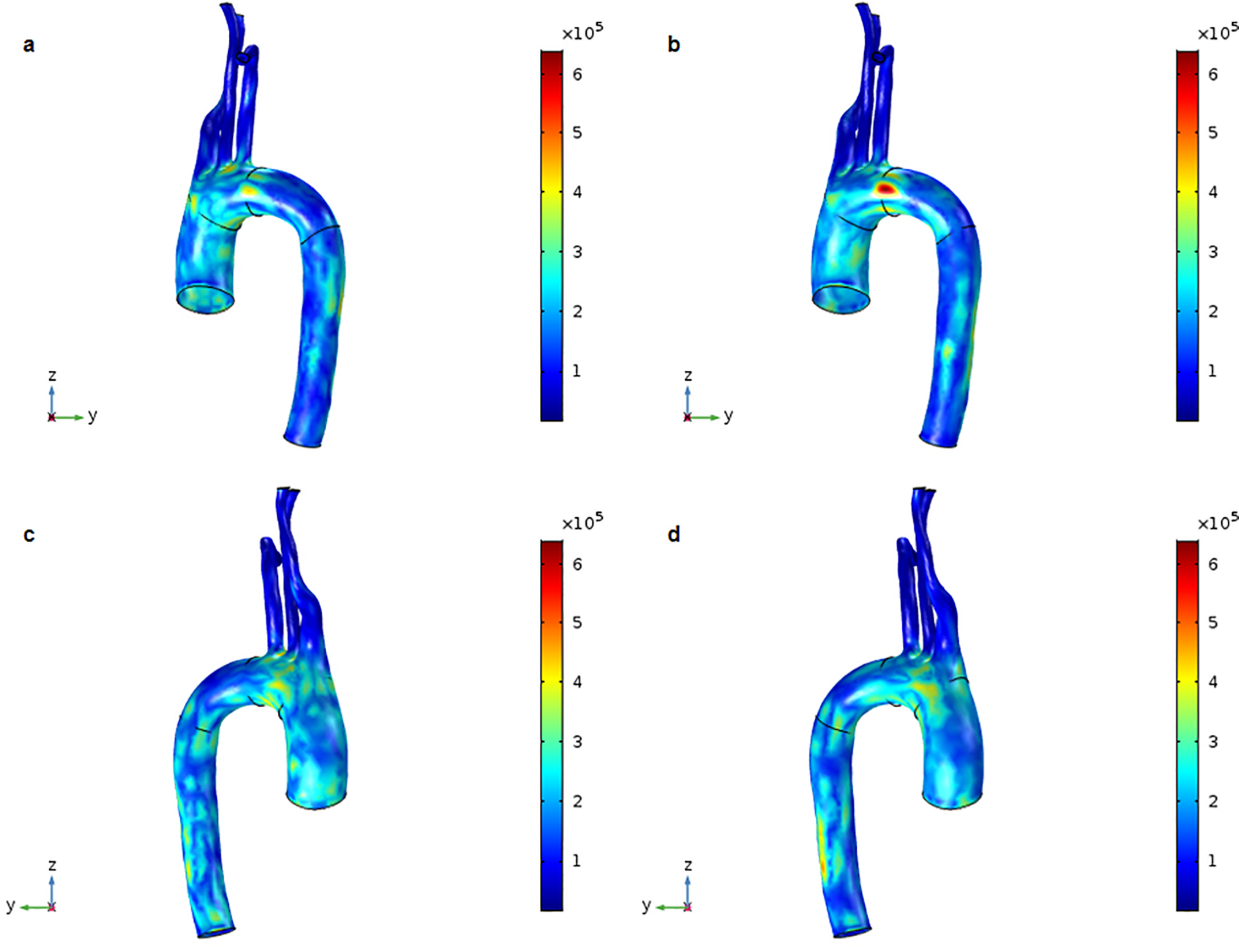
3D distribution of von Mises stress at the systolic peak (t = 0.27 s). (a) Surface values for the control aortic model. (b) Surface values for the DM aortic model. (c) Surface values for the control aortic model other side. (d) Surface values for the DM aortic model other side.

**Fig 7:**
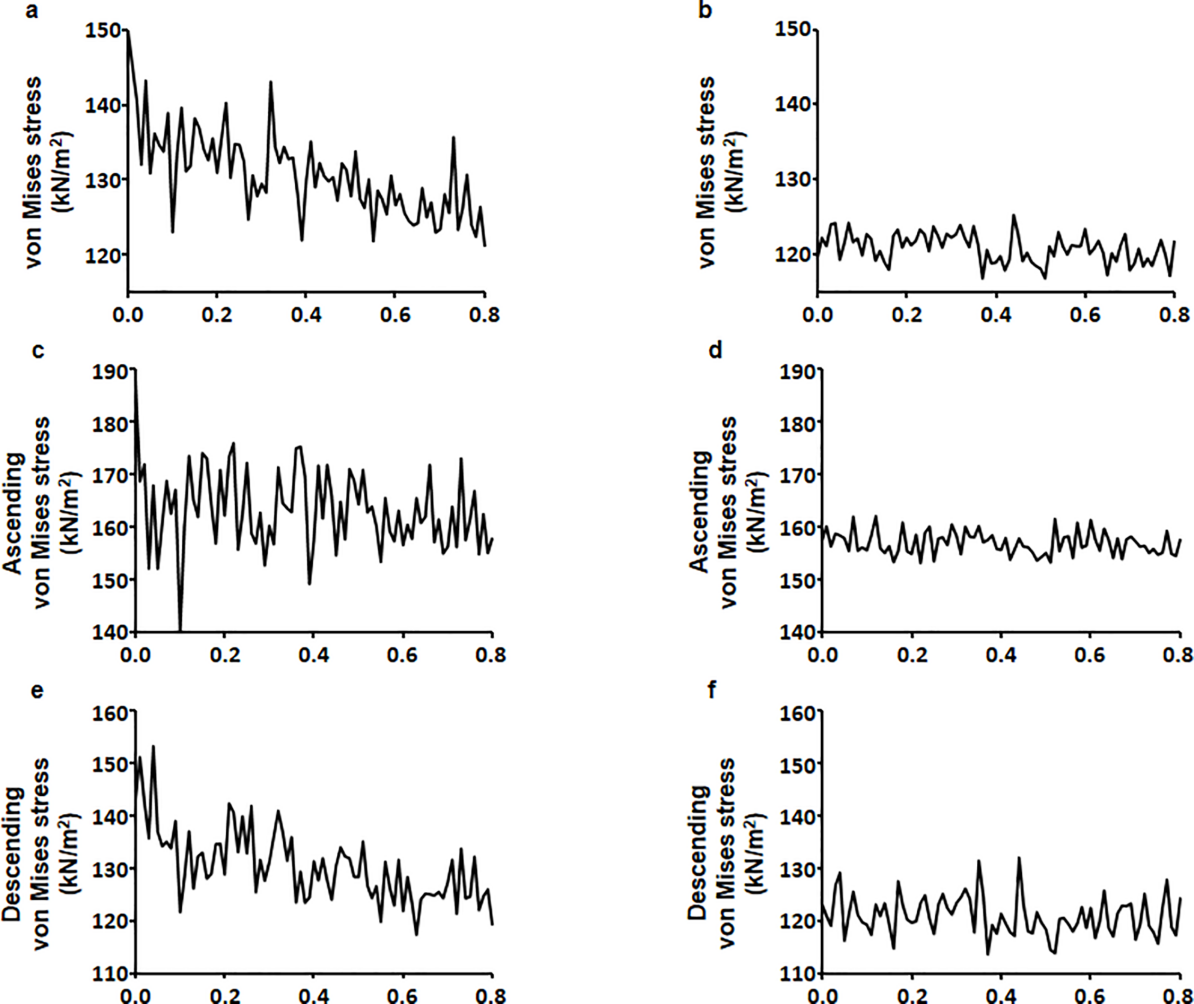
Overall mean von Mises stress. (a) Control and (b) DM aortic models during the cardiac cycle. Ascending aorta von Mises stress for the (c) control and (d) DM aortic models. Descending von Mises stress for the (e) control and (f) DM aortic models.

The measurement of von Mises stress is a standard method of predicting material destruction under force. However, in this study, we used von Mises stress to measure stress range. Control and DM aortic models exhibited higher von Mises stress in the aortic arch than in other sections of the aorta (Fig 6). The maximum movement was 2.051 mm for the control model and 2.150 mm for the DM model, indicating that the control model showed less aortic movement. The maximum von Mises stress values for the control and DM models were 668.454 kN/m^2^ and 647.088 kN/m^2^, respectively. Also, von Mises stress values ranged between 121.028–149.880 kN/m^2^ for the control model and 116.914–125.318 kN/m^2^ for the DM model (Fig 7A and 7B). von Mises stress values for the ascending aorta ranged from 140.445–176.013 kN/m^2^ for the control model and 153.282–161.978 kN/m^2^ for the DM model (Fig 7C and 7D). von Mises stress values for the descending aorta ranged from 117.386–153.103 kN/m^2^ for the control model and 113.857–131.936 kN/m^2^ for the DM model (Fig 7E and 7F).

The results obtained in this study were summarized in Table 1. High pressure and stress in the ascending aorta is known to be involved in diseases such as aneurysm and atherosclerosis [26–28]. We were able to obtain contraction and relaxation information that was not well shown for the DM model due to its stiffness by stress.

**Table 1:** Summary of simulation results for control and DM model.

	Max. movement (mm)	Velocity (m/s)	Pressure (kPa)		von Mises stress (kN/m^2^)		
		Mean	Max.	Min.	Max.	Ascending aorta	Descending aorta
**Control model**	2.051	0.637	17.746	14.558	668.454	140.445–176.013	117.386–153.103
**DM** **model**	2.150	0.585	17.500	14.673	647.088	153.282–161.978	113.857–131.936

## Discussion

This study was undertaken to understand the relationship between biomechanical properties of the aorta and pathologies associated with aortic diseases (e.g., aneurysm, dissection, or atherosclerotic occlusion) using 3D computational aortic models. These models utilized image-based aortic geometry and blood pressure during the cardiac cycle in normal and DM conditions.

Abnormal blood rheology in DM, in particular a greater low blood viscosity, may be related to the increased incidence of macrovascular disease in DM patients [29]. The microstructure of the aortic wall differs depending on the region of the aorta and is altered in DM, which can lead to mechanical and functional changes in vulnerable areas [30–32]. Alterations in aortic hemodynamics have been associated with diabetes [33]. Aortic stiffening is exacerbated by diabetes and changes in aortic stiffness render it more susceptible to disease. This increases morbidity and mortality rates in diabetes patients [34, 35]. Diabetes also results in increased wall stiffening of other arteries such as the common carotid and femoral arteries [36]. In the present study, we examined the effect of DM on biomechanical properties of the aorta by modifying boundary conditions in a 3D aortic model based on human aortic parameters established in previous studies [25, 37]. This aortic model has been used to analyze fluid mechanics using the Navier-stoke equation and treats blood as a Newtonian fluid [38, 39]. Our simulations resulted in flow velocity, pressure, and von Mises stress values that are largely similar to previously reported values [40–42].

A novel aspect of the present work is our detailed analysis of blood flow, pressure, and stress in a DM aortic model using the computational fluid dynamics method, which can be used to predict possible regions where aneurysm, dissection, or atherosclerotic occlusion may occur. We observed higher von Mises stress in the ascending aorta in the control model than in the DM model.

Studies that model the geometrical properties of the human aorta have recognized that aortic diseases are closely associated with changes in hemodynamic properties. Therefore, such models could potentially be used to predict aortic lesions resulting from aortic geometry and pathological conditions. We found that blood flow velocity was faster in the proximal ascending and distal descending aorta and showed a parabolic pattern during the cardiac cycle. These increases in flow velocity may result from the influence of the left ventricular pressure on the proximal ascending aorta and the significant tapering of the descending aortic wall, which is consistent with physiological properties of the aorta [43–45]. The blood pressure profile results from a constant pressure distribution as a function of distance from the left ventricle [46], and the acceleration of blood flow velocity means that maximum pressure is observed at the ascending aorta [47]. Our study shows that the ascending aorta is a high-pressure region, which is consistent with the occurrence of diseases such as aneurysm and atherosclerosis at this location. Thus, the structure of the ascending aorta makes it more susceptible to disease [26–28]. We also examined von Mises stress depending on aortic location. We observed significant von Mises stress in the ascending aorta, resulting in a deformation of the blood vessel due to force.

Computational Fluid Dynamics (CFD) is an attractive tool in hemodynamic analysis, and there have been a lot of discussion on what the results of the CFD study can describe. However, it is still quite unknown how this study will be beneficial in the scientific and medical fields. Our study used the same structural model for normal and diabetic patients, but different results were obtained due to the application of different material properties. Our CFD study is being performed not only in the aorta but also in the carotid and coronary arteries. Patient-specific CFD provides non-invasive functional assessment of the aorta, as well as carotid and coronary arteries integrally, which may be useful in future patient-specific computational models that are used to assess the risk of aortic complications by applying patient-specific characteristics in the cardiovascular system. Moreover, if the results obtained in this study are extended to integrate with molecular biology simulations of endothelial cells, they may play an important role in cardiovascular disease, especially atherosclerosis [45].

There is a research limitation of our study to be addressed here. We implemented only one aortic model for 3D computational analysis, which is not sufficient to perform statistical analysis. Nevertheless, the results of our simulations obtained using the proposed 3D computational models are largely similar to previously reported values [37–39]. The results gained in this study will be strengthened by increasing a sample size and including more parameters. Therefore, more patients will be studied later and statistical analysis will be possible.

## Conclusions

The main aim of this study was to characterize the biomechanical properties of the human aorta under DM conditions. We found that mean blood flow velocity, aortic pressure, and von Mises stress values were lower in the DM aortic model than in the control aortic model. This unique dynamic 3D aortic model approach increases our understanding of aortic abnormalities in DM patients. Thus, when biomechanical aortic properties associated with particular diseases are known, it may be reasonable to use these values in a clinical setting to predict of the risk of adverse aortic events in patients.
